# Evaluating Sex Differences in the Effect of Increased Systolic Blood Pressure on the Risk of Cardiovascular Disease in Asian Populations: A Systematic Review and Meta-Analysis

**DOI:** 10.5334/gh.1159

**Published:** 2022-10-04

**Authors:** Yu-Ting Lin, Yun-Ru Chen, Yu-Chung Wei

**Affiliations:** 1 Institute of Public Health, National Yang Ming Chiao Tung University, Hsinchu, Taiwan; 2 Department of Statistics, Feng Chia University, Taichung, Taiwan; 3 Graduate Institute of Statistics and Information Science, National Changhua University of Education, Chunghua, Taiwan

**Keywords:** cardiovascular health, hypertension, meta-analysis, sex differences, systolic blood pressure

## Abstract

**Background:**

Cardiovascular disease (CVD) is a serious health concern worldwide, and half of the cases of CVD occur in Asia. Because hypertension or high blood pressure has been confirmed to be an important risk factor for CVD, controlling blood pressure is helpful for CVD prevention. Although many studies have shown a sex difference in the impact of blood pressure on the risk of CVD, the risk threshold of blood pressure remained the same for both sexes in the latest global guidelines.

**Objective:**

The study aimed to evaluate sex differences in the effect of increased blood pressure on the risk of CVD in Asian populations.

**Methods:**

In this study, we performed a systematic review via PubMed, Embase, and MEDLINE to select studies conducted with Asian populations published before 30 June 2021.

**Results:**

Six female and eleven male effect sizes for CVD risk from six articles were identified. The unadjusted pooled effect size for CVD risk per 10-mmHg increase in systolic blood pressure was estimated to be 1.20 for females (95% confidence interval: [1.10, 1.32]) and 1.19 for males (95% confidence interval: [1.11, 1.27]). Furthermore, using meta-regression to adjust for the significant effect of smoking, we showed that the impact of a 10-mmHg systolic blood pressure increase on CVD risk among females was 1.232 times that among males, corresponding to a significant sex difference (95% confidence interval: [1.065, 1.426]; *P* = 0.02). In summary, the effect of an increased systolic blood pressure on the risk of CVD in females was significantly higher than that in males in the Asian population.

## Introduction

Cardiovascular disease (CVD) is the leading cause of death among noncommunicable diseases worldwide [1,2,3,4,5,6]. Among the estimated 17.1–19.7 million people worldwide that die from CVDs, approximately 58% of cases occur in Asia, the continent with the largest population in the world [7]. In Asia, approximately 35% of total deaths are caused by CVD, and Asia has a higher proportion of premature CVD death (39%) than that worldwide (34%) [7]. In most Asian countries, the CVD epidemic is rapidly increasing, and CVD deaths are even exceeding cancer deaths in some Asian regions [8]. The number of CVD-related deaths is expected to continue to increase [9]. The burden of CVDs on public health and healthcare systems is significant [10,11]. Reports from the Asia-Pacific region showed that the indirect costs of CVDs associated with long-term functional impairment were even higher than the direct hospitalization and drug costs [12]. The total costs of CVDs have continued to increase and are predicted to increase exponentially [13,14,15].

High blood pressure (BP), or hypertension, is one of the most important risk factors for CVD-related incidence and mortality [4,6,12,16,17,18,19,20,21]. The prevalence of hypertension is 32% in women and 34% in men aged 30 to 79 years worldwide [22]. The average prevalence of hypertension in Asia is similar to the global prevalence [23], but the prevalence in East Asia is higher than that in any other region in Asia [24]. Systolic blood pressure (SBP) is a significant predictor of cardiovascular events [25]. The projected number of individuals with high SBP (≥140 mmHg) doubled from 1990 to 2015, and an estimated 7.8 million deaths were attributed to high SBP in 2015 [26].

BP control has led to a confirmed reduction in cardiovascular events. However, sex differences in the impact of BP on CVDs were not consistent in several previous studies [25,27,28,29,30,31,32,33,34,35,36] and were shown in the Asian population [37,38,39,40]. The mean BP of men is higher than that of women [41,42], and the prevalence of hypertension among males is generally higher than that among females until the age of 60 or even 75 years [43,44,45]. However, abundant research has demonstrated that the impact of increased BP on CVD risk among females is much more severe than that among males [25,46]. However, the latest report from the Eighth Joint National Committee (JNC8) did not provide sex-specific BP treatment guidelines [47].

The prevalence, mortality, and burden of CVD differ among populations due to differences in genetics, environment, culture, and dietary habits [48,49,50]. Epidemiological studies have also revealed that the distribution of CVD risk factors is different among countries [51,52]. Most research and guidelines focus on different ethnic groups in the US population [47,53], but Asia has approximately 60% of the total world population and has rapidly increasing CVD and hypertension epidemics. In this study, we propose a systematic review and meta-analysis to assess the sex effect of SBP on CVD risk specifically in the Asian population. The results provide further research opportunities to develop a suitable prevention program for hypertension to reduce the risk of CVDs.

## Methods

This study followed the Preferred Reporting Items for Systematic Reviews and Meta-Analyses (PRISMA) [54] and the Meta-Analysis of Observational Studies in Epidemiology (MOOSE) [55] guidelines for the literature review and meta-analysis (Supplementary Tables 1 and 2).

### Literature search and review

MEDLINE, Embase and PubMed were used for the literature search. MEDLINE is a bibliographic database containing journal articles in the fields of medical and life sciences from more than 80 countries worldwide. MEDLINE is the primary component of the PubMed and Embase search platforms. PubMed additionally includes online books, life science journals, and articles that have not been formally published, and Embase additionally includes conference proceedings, biomedical science- or medicine-related journals that are not included in PubMed and MEDLINE, as well as many more Eurasian articles.

We searched articles written in English and published before 30 June 2021 through MEDLINE, Embase and PubMed. The search terms were set based on the population-intervention-comparator-outcome (PICO) design of this study. For P, we focused on Asian adults. The C category was the sex subgroup, including males and females. The O category broadly represented the effect of an increase in SBP on CVD risk changes. Because we focused on sex comparisons, no intervention was performed in this study. The search keywords and MeSH terms included cardiovascular disease, heart disease, blood pressure, systolic blood pressure, risk, morbidity, female, male, Asia, and Asian, as well as restricting the study population to adults (Supplementary Table 3). The following criteria were used for screening the titles, abstracts, and articles: (1) the endpoint was CVD; (2) males and females had specific baseline characteristics and CVD outcomes; (3) cross-country studies were allowed, but the subjects were required to be Asian; and (4) the effect size (ES) used to adjust the available risk factors for CVD risk per SBP increment was reported. ESs included the odds ratio (OR)/hazard ratio (HR) and corresponding confidence interval (CI) or standard error (SE). ES with CI or SE between different BP category groups was also allowed. If multiple ESs were provided in an article, the ES that adjusted medication use was prioritized. The data retrieved from the meta-analysis article that matched the above criteria are also available. Duplicate articles were excluded. Two reviewers searched and assessed each article independently, and an advisor then double-checked all identified publications.

The Newcastle-Ottawa Scale was used to determine the study quality (Supplementary Table 4) [56]. Three elements were assessed, including the selection (4 items with 1 point each), comparability (1 item with up to 2 points), and outcome (3 items with 1 point each). The studies were classified based on the total score (0 to 9 points) as good (more than 6 points), fair (4 to 6 points), or poor quality (less than 4 points).

### Data extraction and synthesis

The ESs retrieved from the articles were adjusted HRs, but a few studies reported adjusted ORs instead of adjusted HRs. Because the OR can be considered a time-unit-free index and approximates HR in a population with a low incidence of events [57,58], we treated HR and OR as equivalents, in accordance with other studies [46,59,60].

We adopted the ES in the meta-analysis as the adjusted HR or OR per 10-mmHg SBP increment in CVD risk, which was denoted as ES_10_. However, the unit or type of ES was not consistent for these retrieved publications, and a statistical method was adopted to synthesize ESs. First, if the ES per *k*-mmHg SBP increment was reported (denoted as ES*_k_*), the log-linear transformation was used to adjust it to 10 mmHg [46,61]. The formula is as follows: log(ES_10_) = 10 log(ES*_k_*)/*k*. The SE and lower and upper bounds of CI for ES were converted in the same manner. Second, for the ESs between the two BP groups, the unit of SBP increment was estimated using the mean SBP difference between groups. If the mean SBP was not available but the bounds of the SBP group were provided instead, the midpoint of the SBP group was used. For the data that provided only the upper or lower bound of the SBP interval, the half-width of the adjacent interval was adopted to estimate the mean SBP for the open interval group [46]. Moreover, if multiple ESs that depend on the same BP reference group were adopted from one publication, the ESs and corresponding CI were further adjusted [62]. Then, the log-transformation introduced before was used to estimate the ES_10_.

Moreover, baseline data that were available in all identified articles, such as sample size, sex, age, baseline mean SBP (SBP_B_), and the proportion of smokers among the participants, were collected. Every ES ID was named a string that included gender (M: male; F: female) and study abbreviations. If a study contained more than two ESs, numbers were added at the end of the ES ID. Otherwise, if more than one publication included participants from the same population, the article with the largest sample size was included in the analysis.

### Meta-Analysis

Two statistical approaches were used to assess the sex difference in the association of SBP with CVD risk. First, the sex-specific unadjusted pooled ES was estimated. The random-effect model was adopted with inverse variance weighting for each ES. This approach simply revealed the outcome of each study and summarized the results for gender-specific groups. Second, if other potential risk factors were of concern, a meta-regression analysis was performed to evaluate the significance of the effect of sex on the ES, after adjustment for other available risk factors. The random-effect model with a permutation test approach was adopted for the meta-regression [63,64]. The full model, which included all available risk factors, and the optimal model obtained through the model selection process with the minimum Bayesian information criterion (BIC) are reported.

Cochran’s Q test, the I_2_ statistic, and forest plots were used to evaluate the heterogeneity of the retrieved studies [65,66]. The null hypothesis of Cochran’s Q test assumed that the effectiveness of the treatments in all studies was equal [67]. I_2_ values (0–100%) of 25%, 50%, and 75% indicated low, medium, and high degrees of heterogeneity, respectively [68].

In addition, potential publication bias was assessed using Begg’s and Mazumdar’s rank correlation test (Begg’s test) [69] and Egger’s regression test (Egger’s test) [70]. A funnel plot was also used to visualize the bias. Leave-one-out sensitivity analysis was used to evaluate the influence of the specific ES on the overall results. If necessary, extended sensitivity analyses for subsets of ESs were performed for further discussion. All analyses were performed using the *dosresmeta* [71] and *metafor* [72] packages in R software.

## Results

### Literature search and reviews

**Figure 1:**
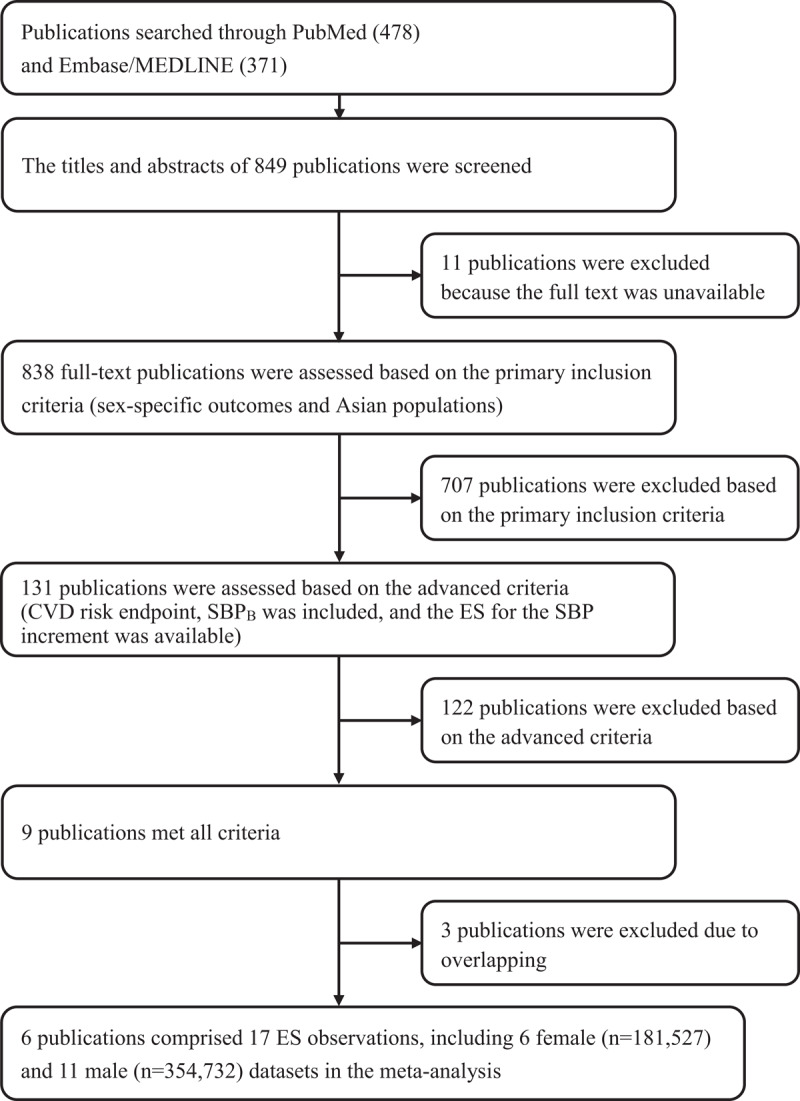
Flow chart of the publication selection. CVD: cardiovascular disease; ES: effect size; HR: hazard ratio; SBP: mean systolic blood pressure; SBP_B_: mean systolic blood pressure at baseline.

A total of 849 publications were searched through PubMed (478) and Embase/MEDLINE (371). Finally, six female and eleven male ESs from six publications that met the search criteria were included [39,73,74,75,76,77]. A flow chart of the literature search and review is shown in Figure 1. The summary information for these retrieved articles is listed in Table 1 and Supplementary Table 5. All studies were considered to have good quality according to the quality assessment criteria (Supplementary Table 4).

**Table 1:** Characteristics of the selected studies.


SEX	STUDY*	ID	SAMPLE SIZE	ES	REPORTED ES [95% CI]^†^	UNIT (mmHg)	SBP_B_(mmHg)	AGE (YEAR)	SMOKING (%)

***Female***	APCSC [73]	F-APCSC	168910	HR	1.38 [1.25, 1.51]	10	119	42	5

	PREDICT [74]	F-PREDICT	7039	HR	1.23 [1.12, 1.36]	10	126.1	52.9	1

	CONOR [74]	F-CONOR	967	HR	1.06 [0.85, 1.31]	10	119.1	40.3	1

	SUITA [75]	F-SUITA-1	504	HR	1.17 [0.32, 4.34]	16.9	122.4	54	11.7

		F-SUITA-2	465	HR	1.83 [0.58, 5.75]	26.9	132.4	58.9	9.2

	TLGS [76]	F-TLGS	3642	HR	1.01 [1.004, 1.015]	1	122	46.5	3.6

***Male***	APCSC [73]	M-APCSC	331909	HR	1.38 [1.28, 1.46]	10	122	45	59

	JPHC [39]	M-JPHC-1	2456	HR	1.35 [0.63, 2.88]	10	125	53.1	45.9

		M-JPHC-2	2483	HR	1.32 [0.62, 2.80]	20	135	54.6	44.6

		M-JPHC-3	3371	HR	1.98 [0.99, 3.98]	35	150	56	43.2

		M-JPHC-4	1028	HR	2.69 [1.23, 5.89]	55	170	56.5	40.3

		M-JPHC-5	288	HR	3.74 [1.47, 9.53]	75	190	56.3	44.5

	PREDICT [74]	M-PREDICT	9997	HR	1.03 [0.96, 1.11]	10	125.3	47.4	9

	CONOR [74]	M-CONOR	1239	HR	1.14 [0.98, 1.32]	10	126.6	41.4	25

	SESSA [77]	M-SESSA	996	OR	1.21 [1.03, 1.43]	19	136.1	64	32.2

	SUITA [75]	M-SUITA-1	502	HR	1.78 [0.75, 4.22]	13.9	121.7	54	49.6

		M-SUITA-2	463	HR	2.32 [1.02, 5.27]	23.6	131.4	57.5	46.3


CI: confidence interval; ES: effect size; HR: hazard ratio; ID: identification; OR: odds ratio; SBP_B_: mean systolic blood pressure at baseline; Smoking: the proportion of smokers (APCSC, CONOR, JPHC, PREDICT, SESSA, and SUITA represent current smokers; TLGS represents current and former smokers). * Abbreviations of study names: APCSC: Asia Pacific Cohort Studies Collaboration; PREDIECT: PREDICT-CVD Cohort Study; CONOR: Cohort of Norway, including Oslo Health Study, Oslo Immigrant Health Study, and The Romsås in Motion Study; SUITA: The Suita Study; TLGS: The Tehran Lipid and Glucose Study; JPHC: Japan Public Health Center-based Prospective Study; SESSA: Shiga Epidemiological Study of Subclinical Atherosclerosis. ^†^ The reported ES and corresponding CI were further adjusted if multiple ESs that depend on the same BP reference group were adopted from one publication.

### Meta-Analysis

**Figure 2:**
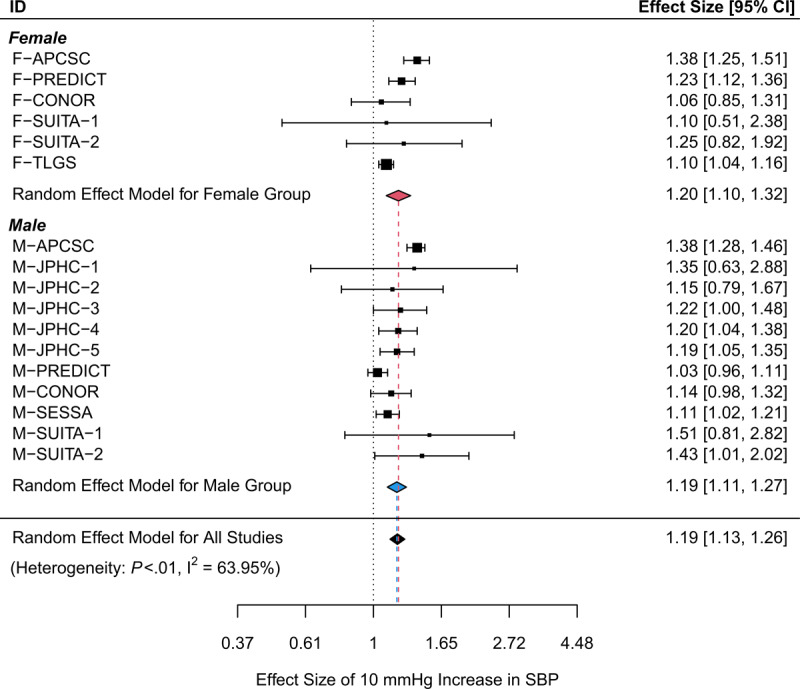
Observed and estimated ESs for the CVD risk per 10-mmHg increase in SBP in Asia.

Based on all the collected data, the pooled ES for CVD risk per 10-mmHg increase in SBP was estimated to be 1.19 (95% CI: [1.13, 1.26]), which indicated that for each 10-mmHg increment in SBP, the CVD risk increased by 19%. In the sex subgroup analysis, the pooled ES for females was 1.20 (95% CI: [1.10, 1.32]), and the pooled ES of males was 1.19 (95% CI: [1.11, 1.27]), which indicated that per 10-mmHg increase in SBP, the CVD risk increased by 20% for females and 19% for males, so females had a higher CVD risk than males. However, the 95% CIs for these two subgroups overlapped, so the pooled ESs of the males and females were not significantly different in the absence of other risk factors. The forest plot in Figure 2 shows the ES of each study and estimated pooled ESs. I_2_ (63.95%) and Cochran’s Q test showed that the heterogeneity was significant (*P* < 0.01). This finding indicates that there may be other potential moderator variables that impact the analysis results. Therefore, multiple meta-regressions adjusted for potential risk factors were used to evaluate the sex effect of the impact of SBP on CVD risk.

The potential moderator variables without missing data, including sex, mean SBP at baseline, age, and the proportion of smokers, were used for the meta-regression. In the full model, sex and the proportion of smokers were significant moderators, but baseline SBP and age were not. In the optimal model, two significant variables, sex and the proportion of smokers, were retained. The BIC was reduced to –19.936 in the optimal model compared with –14.987 in the full model. The results revealed that the risk of CVD per 10-mmHg increase in SBP among females was significantly higher than that among males, and the risk among females was 1.232 times higher than that among males. In addition, the risk of CVD among smokers was 1.006 times that among nonsmokers. I_2_ in the optimal model was reduced to a level indicating moderate heterogeneity (40.03%; 95% CI: [0.00%, 56.38%]). The detailed information is shown in Table 2.

**Table 2:** Moderator estimators for CVD risk in Asian populations via meta-regression.


MODEL	MODERATOR	e^β^	95% CI	*P*

Optimal	Sex (1: female; 0: male)	1.232	[1.065, 1.426]	0.02^†^

	Smoking (%)	1.006	[1.003, 1.009]	<0.01^†^

Full	Sex (1: female; 0: male)	1.212	[1.039, 1.413]	0.03^†^

	Smoking (%)	1.006	[1.003, 1.010]	<0.01^†^

	SBP_B_ (mmHg)	0.999	[0.996, 1.002]	0.72

	Age (years)	0.998	[0.990, 1.006]	0.66


CI: confidence interval; SBP_B_: mean systolic blood pressure at baseline; Smoking: the proportion of smokers. ^†^ Significant (*P* < 0.05).

**Figure 3:**
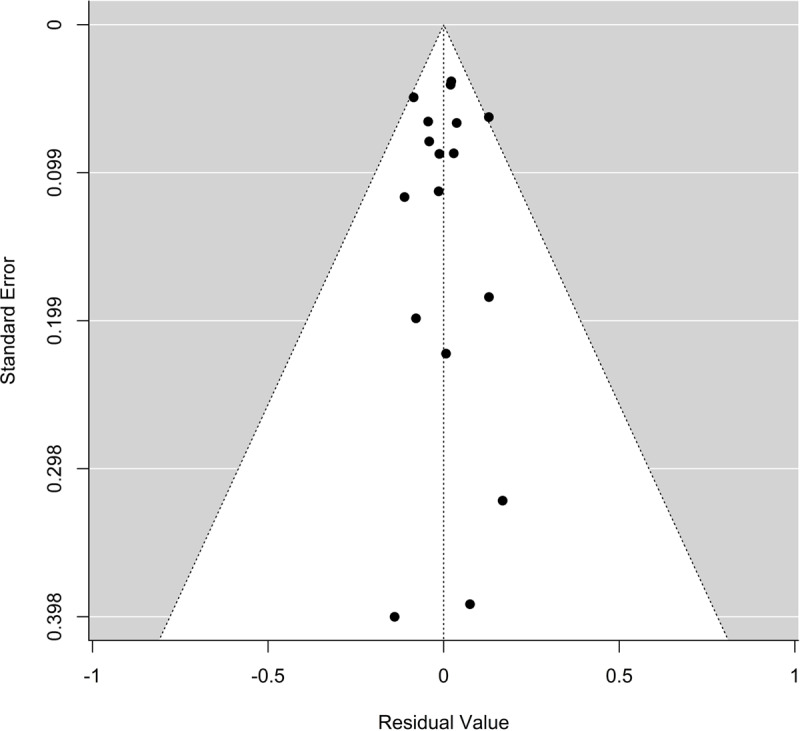
Funnel plot of the publication bias for effect sizes in Asia.

Figure 3 shows that the funnel plot was symmetrical, and only one ES observation fell on the 95% CI bands (F-APCSC). The results of the funnel plot asymmetry testing were also not significant (Begg’s test *P* = 0.49; Egger’s test *P* = 0.87), which indicates an absence of publication bias in this study. The leave-one-out sensitivity analysis revealed no influential observations.

### Further considerations based on sensitivity analysis

For further considerations, the results of specific and additional sensitivity analyses were discussed. The ESs included the HR and OR, which were treated equivalently in our study, as performed in other research [46,59,60]. Because only one ES was reported as an OR from the SESSA study [77] in our retrieved data, the sensitivity analysis result that removed this ES was specifically mentioned. The findings were similar to the results of all the retrieved ESs. The unadjusted pooled ES did not show a significant sex-related difference (Supplementary Figure 1). The optimal meta-regression model still showed that females were 1.216 (95% CI: [1.044, 1.416]) times more likely than males to have a significant CVD risk per 10-mmHg SBP increment. The significant moderator, the proportion of smokers, was also kept at e_β_= 1.006 (95% CI: [1.002, 1.009]). The results are summarized in Supplementary Table 6. The I_2_ in the optimal model was 37.91%, with a 95% CI of [0.00%, 56.16%]. The funnel plot in Supplementary Figure 2 shows one ES observation outside of but closer to the 95% CI bands (F-APCSC) with no significant asymmetry (Begg’s test *P* = 0.89; Egger’s test *P* = 0.94).

Moreover, the CVD data from western, central and southern Asia are limited [24]; only one publication from India/South Asia [74] and one ES from Iran [76] were included in our study. Thus, we performed an additional meta-analysis and meta-regression in which the two publications from India/South Asia and Iran were excluded. The pooled ES for CVD risk per 10-mmHg increase in SBP was estimated to be 1.26 (95% CI: [1.18, 1.34]), which is slightly higher than the results of all the retrieved ESs from Asia (1.19; 95% CI: [1.13, 1.26]). For the independent analyses for both sexes, the pooled ES for females (1.36; 95% CI: [1.21, 1.53]) was higher than that for males (1.24; 95% CI: [1.15, 1.33]), but the difference was not significant. The individual ESs and estimated pooled ESs are also shown in the forest plot (Supplementary Figure 3). Meta-regression was also adopted to adjust for risk factors, including sex, mean SBP at baseline, age, and the proportion of smokers. The optimal model revealed that sex (e_β_ = 1.528; 95% CI: [1.176, 1.984]) and the proportion of smokers (e_β_ = 1.008; 95% CI: [1.003, 1.014]) were significant moderators. This finding indicates that females were 1.528 times more likely than males to have a risk of CVD per 10-mmHg increase in SBP. I_2_ in the optimal model was reduced to 13.60%, indicating low heterogeneity. The detailed information is shown in Supplementary Table 7. The results from the funnel plot (Supplementary Figure 4) and asymmetry test (Begg’s test *P* = 0.74; Egger’s test *P* = 0.96) indicated the absence of publication bias in this study. Regarding the results from pooled ESs and meta-regression, the sex difference remained despite exclusion of the India/South Asia and Iran datasets. However, the sex difference was slightly larger when we considered only these ESs without the India/South Asia and Iran publications.

## Discussion

Sex differences related to CVD are considered an important issue [46,78] but are still underreported in the literature [79]. Assessing sex differences in the impact of BP on CVD risk is valuable for creating health policies related to CVD prevalence and for improving health care. Although some cohort studies and meta-analyses have confirmed the existence of sex differences, the latest guideline from the JNC8 still maintained the same BP treatment guidelines for both sexes. Moreover, because hypertension is more common in East Asia than in any other region in Asia [24], some countries implemented prevention programs for hypertension to improve the CVD incidence several decades ago [80,81]. In Japan, the mean SBP was decreased by 5 mmHg in men and 10 mmHg in women older than 30 years from 1961 to 2000 [82]. Deaths from CVDs in Japan accounted for 40% of all deaths in 1980 and 25% in 2011 [24]. The CVD mortality in South Korea decreased by 57% in men and 48% in women from 1984 to 1999 [83]. The trend in Singapore was similar to those in Japan and South Korea [84]. Based on these longitudinal observational studies, prevention strategies for elevated SBP in CVD patients had a different effect on females and males, which indicated that sex-specific BP guidelines should be considered. The findings of our meta-regression revealed that women have a 1.232-fold higher CVD risk per 10-mmHg SBP increment than men. This finding implied that the elevated SBP threshold for females would be significantly lower than that for males under the same CVD risk conditions. It is reasonable to construct sex-specific BP targets for CVD risk control and decrease sex differences in seeking treatment and sources of care for the Asian population [85].

In our meta-regression, the proportion of smokers was also a significant moderator of the impact of SBP on CVD risk. Smoking seems to be an independent risk factor for coronary heart disease and may have a multiplicative effect on other factors [86]. Smoking, similar to BP, was included as one of the seven core health behaviors or factors associated with CVD [17]. In particular, having two or more major risk factors has been shown to increase the lifetime risk of CVD events by approximately 38.5% to 49.5% in males and approximately 29.2% to 38.7% in females [87]. Prevention programs for both hypertension and smoking have led to CVD event control in Japan [24].

Our study is not without limitations. Biases due to the inconsistency of the considered risk factors, variable definition, participant characteristics, and study design between the studies render the construction of a systematic review and meta-analysis study difficult. First, the baseline SBP and age were not identified as significant in our findings, although they generally were considered important risk factors for CVD. The possible reasons were that the data retrieved from the population that matched the systematic review criteria had similar distributions of baseline SBP and age. Most of our retrieved studies reported a baseline SBP ranging from approximately 120 to 130 mmHg, and only a few studies provide the SBP range at other intervals. Moreover, the studies we retrieved reported a mean age of only approximately 40 to 60 years. In general, the CVD risk associated with SBP would not have significant variance in this narrow age range. If the data were obtained from adults with a wider age range of young to menopausal adults, the age effect would be worth considering. Second, some potential risk factors for CVD were not included in the meta-regression model because they were unavailable in some collected articles, such as diabetes, blood cholesterol levels, and obesity. Nevertheless, the important risk factors were adjusted according to the characteristics of the participants in each study to estimate the ES, which was adopted in our study (Supplementary Table 5). It is noteworthy that a small proportion of persons taking antihypertensive drugs in a few studies was not adjusted to estimate the ES. However, each analysis that adopted the retrieved ES further evaluated the heterogeneity via the I_2_ statistic and Cochran’s Q test, and the moderate level of heterogeneity makes our conclusion convincing. In addition, we concluded that a smoker had a significantly higher risk of CVD with increased SBP than a nonsmoker via meta-regression. Although a slight discrepancy in the definition of smokers was identified between the TLGS study [76] and other studies, our leave-one-out sensitivity analysis revealed that the conclusion was retained.

Moreover, marked variations in race, ethnicities, cultures, socioeconomic status, and geographical region with regard to hypertension and CVD events were observed [88]. Although we limited the study population to the Asian population in our study, the distribution of CVD risk among Asian populations may be different [51,52]. In our study, most ESs were obtained from East Asia, and Japan was the most common country for research studies. This may be because Japan has more advanced medical standards and technology, and the research budget in the field of biomedicine is higher than that in other Asian regions. The analysis results are still valuable because the data from East Asia and Japan may represent the possible future in many Asian countries with regard to population aging, economic development, lifestyle, and epidemiological transition.

Furthermore, to retain the statistical power of the study, we kept as many usable ESs from the retrieved publications as possible, despite inconsistent data issues. First, the relative risk ratio of women to men is a straightforward approach to compare the sex differences in CVDs [89]. However, all studies for which this approach is adopted must include both sexes to derive the relative risk ratio for each study. Unfortunately, the number of studies examining CVD in the Asian population that matched our current search criteria limited the application of this approach. Second, most of the ESs used in our analysis were retrieved from independent studies, but one female ES and one male ES were gathered from the Asia Pacific Cohort Studies Collaboration (APCSC), which was attributed to the meta-analysis of more than thirty publications. We reviewed all the collected data cited from the APCSC study, but none of the articles matched our search criteria due to missing adjusted ESs for SBP increments. For this reason, we had no alternative but to use the summary results from the meta-analysis that included information from many populations.

## Conclusions

In this research, we performed a systematic review and meta-analysis to evaluate the sex differences in the effect of increased SBP on CVD risk in the Asian population. Through the systematic review, six female and eleven male ESs for CVD risk from six articles were identified. Using meta-regression to adjust for the significant effect, we showed that the impact of a 10-mmHg SBP increase on CVD risk among females was 1.232 times that among males, which corresponded to a significant sex difference (95% CI: [1.065, 1.426]; *P* = 0.02) in the Asian population. Based on our findings, it is reasonable to consider sex-specific BP targets for CVD risk assessment and formulate a sex-equivalent health treatment strategy for the Asian population in future studies.

## Additional File

The additional file for this article can be found as follows:

10.5334/gh.1159.s1 Supplementary Material. Supplementary Table 1 to 7 and Supplementary Figure 1 to 4.
