# Supplementary material for: Evaluating Sex Differences in the Effect of Increased Systolic Blood Pressure on the Risk of Cardiovascular Disease in Asian Populations: A Systematic Review and Meta-Analysis
Source: Glob Heart. 2022 Oct 4;17(1):70. doi: 10.5334/gh.1159 (PMC9541122; doi:10.5334/gh.1159)
Supplement: Supplementary Material. — Supplementary Table 1 to 7 and Supplementary Figure 1 to 4. [file gh-17-1-1159-s1.pdf]

Supplementary Material

Evaluating Sex Differences in the Effect of Increased Systolic Blood Pressure on the Risk of Cardiovascular Disease in Asian Populations: A Systematic Review and Meta-Analysis

YU-TING LIN[1], YUN-RU CHEN[2], and YU-CHUNG WEI[3,*]

[1]Institute of Public Health, National Yang Ming Chiao Tung University, Hsinchu, Taiwan.
orcid.org/0000-0003-1975-9749

[2] Department of Statistics, Feng Chia University, Taichung, Taiwan.

[3] ([*]Corresponding author)
Graduate Institute of Statistics and Information Science, National Changhua University of Education, Chunghua, Taiwan.
orcid.org/0000-0003-3747-2215
E-mail: weiyuchung@cc.ncue.edu.tw

Supplementary Table 1. PRISMA checklist

Section/ topic	#	Checklist item	Reported on page #
**TITLE**			
Title	1	Identify the report as a systematic review, meta-analysis, or both.	1
**ABSTRACT**			
Structured summary	2	Provide a structured summary including, as applicable: background; objectives; data sources; study eligibility criteria, participants, and interventions; study appraisal and synthesis methods; results; limitations; conclusions and implications of key findings; systematic review registration number.	1
**INTRODUCTION**			
Rationale	3	Describe the rationale for the review in the context of what is already known.	2
Objectives	4	Provide an explicit statement of questions being addressed with reference to participants, interventions, comparisons, outcomes, and study design (PICOS).	2-3
**METHODS**			
Protocol and registration	5	Indicate if a review protocol exists, if and where it can be accessed (e.g., Web address), and, if available, provide registration information including registration number.	9
Eligibility criteria	6	Specify study characteristics (e.g., PICOS, length of follow-up) and report characteristics (e.g., years considered, language, publication status) used as criteria for eligibility, giving rationale.	2-3
Information sources	7	Describe all information sources (e.g., databases with dates of coverage, contact with study authors to identify additional studies) in the search and date last searched.	2
Search	8	Present full electronic search strategy for at least one database, including any limits used, such that it could be repeated.	3, Supplementary Table 3
Study selection	9	State the process for selecting studies (i.e., screening, eligibility, included in systematic review, and, if applicable, included in the meta-analysis).	3
Data collection process	10	Describe method of data extraction from reports (e.g., piloted forms, independently, in duplicate) and any processes for obtaining and confirming data from investigators.	3
Data items	11	List and define all variables for which data were sought (e.g., PICOS, funding sources) and any assumptions and simplifications made.	3
Risk of bias in individual studies	12	Describe methods used for assessing risk of bias of individual studies (including specification of whether this was done at the study or outcome level), and how this information is to be used in any data synthesis.	3-4
Summary measures	13	State the principal summary measures (e.g., risk ratio, difference in means).	3-4
Synthesis of results	14	Describe the methods of handling data and combining results of studies, if done, including measures of consistency (e.g., I^2) for each meta-analysis.	3-4
Risk of bias across studies	15	Specify any assessment of risk of bias that may affect the cumulative evidence (e.g., publication bias, selective reporting within studies).	4
Additional analyses	16	Describe methods of additional analyses (e.g., sensitivity or subgroup analyses, meta-regression), if done, indicating which were pre-specified.	3-4

Section/ topic	#	Checklist item	Reported on page #
**RESULTS**			
Study selection	17	Give numbers of studies screened, assessed for eligibility, and included in the review, with reasons for exclusions at each stage, ideally with a flow diagram.	4, Figure 1
Study characteristics	18	For each study, present characteristics for which data were extracted (e.g., study size, PICOS, follow-up period) and provide the citations.	4, Table 1, Supplementary Table 5
Risk of bias within studies	19	Present data on risk of bias of each study and, if available, any outcome level assessment (see item 12).	5-7, Figure 3, Supplementary Figure 2, Supplementary Figure 4
Results of individual studies	20	For all outcomes considered (benefits or harms), present, for each study: (a) simple summary data for each intervention group (b) effect estimates and confidence intervals, ideally with a forest plot.	5-7, Figure 2, Supplementary Figure 1, Supplementary Figure 3
Synthesis of results	21	Present results of each meta-analysis done, including confidence intervals and measures of consistency.	5-7, Figure 2, Supplementary Figure 1, Supplementary Figure 3
Risk of bias across studies	22	Present results of any assessment of risk of bias across studies (see Item 15).	5-7, Figure 3, Supplementary Figure 2, Supplementary Figure 4
Additional analysis	23	Give results of additional analyses, if done (e.g., sensitivity or subgroup analyses, meta-regression [see Item 16]).	5-7, Figure 2, Table 2, Supplementary Figure 1, Supplementary Figure 3, Supplementary Table 6-7
**DISCUSSION**			
Summary of evidence	24	Summarize the main findings including the strength of evidence for each main outcome; consider their relevance to key groups (e.g., healthcare providers, users, and policy makers).	7-8
Limitations	25	Discuss limitations at study and outcome level (e.g., risk of bias), and at review-level (e.g., incomplete retrieval of identified research, reporting bias).	7-8
Conclusions	26	Provide a general interpretation of the results in the context of other evidence, and implications for future research.	9
**FUNDING**			
Funding	27	Describe sources of funding for the systematic review and other support (e.g., supply of data); role of funders for the systematic review.	9

From reference [1]: **Moher D, Liberati A, Tetzlaff J, Altman DG.** Preferred reporting items for systematic reviews and meta-analyses: the PRISMA statement. *International Journal of Surgery*. 2010; 8(5): 336-41. DOI: https://doi.org/10.1016/j.ijsu.2010.02.007

Supplementary Table 2. MOOSE checklist

Item No.	Recommendation	Reported on Page No.
Reporting of background should include		
1	Problem definition	2
2	Hypothesis statement	2
3	Description of study outcome(s)	3
4	Type of exposure or intervention used	3
5	Type of study designs used	NA
6	Study population	2-3
Reporting of search strategy should include		
7	Qualifications of searchers (e.g., librarians and investigators)	3
8	Search strategy, including time period included in the synthesis and key words	2-3, Figure 1, Supplementary Table 3
9	Effort to include all available studies, including contact with authors	2-3
10	Databases and registries searched	2
11	Search software used, name and version, including special features used (e.g., explosion)	2
12	Use of hand searching (e.g., reference lists of obtained articles)	Table 1, Supplementary Table 5
13	List of citations located and those excluded, including justification	2-3, Figure 1
14	Method of addressing articles published in languages other than English	NA
15	Method of handling abstracts and unpublished studies	3, Figure 1
16	Description of any contact with authors	NA
Reporting of methods should include		
17	Description of relevance or appropriateness of studies assembled for assessing the hypothesis to be tested	3-4
18	Rationale for the selection and coding of data (e.g., sound clinical principles or convenience)	3-4
19	Documentation of how data were classified and coded (e.g., multiple raters, blinding and interrater reliability)	3
20	Assessment of confounding (e.g., comparability of cases and controls in studies where appropriate)	3-4
21	Assessment of study quality, including blinding of quality assessors, stratification or regression on possible predictors of study results	3-4
22	Assessment of heterogeneity	4
23	Description of statistical methods (e.g., complete description of fixed or random effects models, justification of whether the chosen models account for predictors of study results, dose-response models, or cumulative meta-analysis) in sufficient detail to be replicated	3-4
24	Provision of appropriate tables and graphics	Figure 1-3, Table 1-2, Supplementary Figure 1-4, Supplementary Table 3-7

Item No.	Recommendation	Reported on Page No.
Reporting of results should include		
25	Graphic summarizing individual study estimates and overall estimate	Figure 2,<br>Supplementary Figure 1,<br>Supplementary Figure 3
26	Table giving descriptive information for each study included	Table 1,<br>Supplementary Table 5
27	Results of sensitivity testing (e.g., subgroup analysis)	5-7, Figure 2,<br>Supplementary Figure 1,<br>Supplementary Figure 3
28	Indication of statistical uncertainty of findings	5, 7
Reporting of discussion should include		
29	Quantitative assessment of bias (e.g., publication bias)	5-8, Figure 3,<br>Supplementary Figure 2,<br>Supplementary Figure 4
30	Justification for exclusion (e.g., exclusion of non-English language citations)	3-4, 8, Figure 1
31	Assessment of quality of included studies	4<br>Supplementary Table 4
Reporting of conclusions should include		
32	Consideration of alternative explanations for observed results	7,<br>Supplementary Figure 1-4,<br>Supplementary Table 6-7
33	Generalization of the conclusions (i.e., appropriate for the data presented and within the domain of the literature review)	9
34	Guidelines for future research	7-9
35	Disclosure of funding source	9

From reference [2]: **Stroup DF, Berlin JA, Morton SC, Olkin I, Williamson GD, Rennie D,** et al. Meta-analysis of observational studies in epidemiology: a proposal for reporting. *JAMA*. 2000; 283(15): 2008-12. DOI: https://doi.org/10.1001/jama.283.15.2008

Supplementary Table 3. Search strategies for the literature review

Database	Search terms	Date	Records
PubMed	("cardiovascular diseases"[MeSH Terms] OR "heart disease"[All Fields]) AND ("blood pressure"[MeSH Terms] OR "blood pressure"[All Fields]) AND ("risk"[MeSH Terms]) AND ("sex factors"[MeSH Terms] OR "sex distribution"[MeSH Terms] OR "sex"[All Fields] OR "Women’s Health"[MeSH Terms]) AND ("female"[MeSH Terms] OR "male"[MeSH Terms]) AND ("aged, 80 and over"[MeSH Terms] OR "adult"[MeSH Terms] OR "young adult"[MeSH Terms] OR "adult"[MeSH Terms:noexp] OR ("middle aged"[MeSH Terms] OR "aged"[MeSH Terms]) OR "middle aged"[MeSH Terms] OR "aged"[MeSH Terms]) AND ("humans"[MeSH Terms]) AND English[lang] AND ("0001/01/01"[PDAT]: "2021/06/30"[PDAT]) AND ("asia" OR "asian")	30 June 2021	478
Embase/ MEDLINE	('cardiovascular disease'/mj OR 'heart disease'/exp) AND ('blood pressure'/de OR 'systolic blood pressure'/de) AND ('risk'/exp OR 'morbidity'/de) AND ('female'/exp OR 'male'/exp) AND ([young adult]/lim OR [adult]/lim OR [middle aged]/lim OR [aged]/lim OR [very elderly]/lim) AND [humans]/lim AND [1-1-1945]/sd NOT [30-06-2021]/sd AND [english]/lim AND [article]/lim AND ('asia' OR 'asian')	30 June 2021	371

Supplementary Table 4. Newcastle–Ottawa scales for quality assessment of the included studies

Study*	Selection	Comparability	Outcome	Total
APCSC [3]	3	2	3	8
PREDICT & CONOR [4]	3	2	3	8
SUITA [5]	3	2	3	8
TLGS [6]	3	2	3	8
JPHC [7]	3	2	3	8
SESSA [8]	3	2	2	7

*****Abbreviations of study names: APCSC: Asia Pacific Cohort Studies Collaboration; PREDIECT: PREDICT-CVD Cohort Study; CONOR: Cohort of Norway, including Oslo Health Study, Oslo Immigrant Health Study, and The Romsås in Motion Study; SUITA: The Suita Study; TLGS: The Tehran Lipid and Glucose Study; JPHC: Japan Public Health Center-based Prospective Study; SESSA: Shiga Epidemiological Study of Subclinical Atherosclerosis.

Supplementary Table 5. Summary of retrieved studies in the meta-analysis

Sex	Study*	Disease[†]	Country/Population	ES	Design	Adjusted variable(s)
Female	APCSC [3]	IHD	China, Hong Kong, Japan, South Korea, Taiwan, Singapore, Thailand	HR	prospective cohort	Age, BMI, Total cholesterol, Triglycerides, Smoking
	PREDICT [4]	CVD	India (living in New Zealand)	HR	prospective cohort	Age, Blood pressure medication use
	CONOR [4]	CVD	South Asia (living in Norway)	HR	prospective cohort	Age, Blood pressure medication use
	SUITA [5]	MI	Japan	HR	prospective cohort	Age, BMI, Hyperlipidemia, Diabetes, Smoking, Drinking status
	TLGS [6]	CVD	Iran	HR	prospective cohort	Age, Total cholesterol, Fasting plasma glucose, Smoking, Family history of CVD, Wrist circumference
Male	APCSC [3]	IHD	China, Hong Kong, Japan, South Korea, Taiwan, Singapore, Thailand	HR	prospective cohort	Age, BMI, Total cholesterol, Triglycerides, Smoking
	JPHC [7]	CHD	Japan	HR	prospective cohort	Age, BMI, Smoking status, Ethanol intake, Antihypertensive medication use, Diabetes, Serum total cholesterol levels, Public health center areas
	PREDICT [4]	CVD	India (living in New Zealand)	HR	prospective cohort	Age, Blood pressure medication use
	CONOR [4]	CVD	South Asia (living in Norway)	HR	prospective cohort	Age, Blood pressure medication use
	SESSA [8]	CAC	Japan	OR	cross-sectional	Age, BMI, Smoking, Antihypertensive use, HDL-C, Total cholesterol, Dyslipidemia medication use, Diabetes, eGFR, CT-type (EBCT/16-MDCT)
	SUITA [5]	MI	Japan	HR	prospective cohort	Age, BMI, Hyperlipidemia, Diabetes, Smoking, Drinking status

16-MDCT: 16-channel multidetector row computed tomography; BMI: body mass index; EBCT: electron beam computed tomography; eGFR: estimated glomerular filtration rate; ES: effect size; HDL-C: high-density lipoprotein cholesterol; HR: hazard ratio; OR: odds ratio.

***Abbreviations of study names: APCSC: Asia Pacific Cohort Studies Collaboration; PREDIECT: PREDICT-CVD Cohort Study; CONOR: Cohort of Norway, including Oslo Health Study, Oslo Immigrant Health Study, and The Romsås in Motion Study; SUITA: The Suita Study; TLGS: The Tehran Lipid and Glucose Study; JPHC: Japan Public Health Center-based Prospective Study; SESSA: Shiga Epidemiological Study of Subclinical Atherosclerosis.

[†]Abbreviations of diseases: CAC: coronary artery calcification; CHD: coronary heart disease; CVD: cardiovascular disease; IHD: ischemic heart disease; MI: myocardial infarction.

Supplementary Table 6. Moderator estimators for CVD risk in Asia (excluding the SESSA study)

Model	Moderator	e^{β}	95% CI	*P*
Optimal	Sex (1: female; 0: male)	1.216	[1.044, 1.416]	0.03‡
	Smoking (%)	1.006	[1.002, 1.009]	<0.01‡
Full	Sex (1: female; 0: male)	1.201	[1.025, 1.407]	0.06
	Smoking (%)	1.006	[1.002, 1.010]	<0.01‡
	SBP_B (mmHg)	0.999	[0.995, 1.002]	0.60
	Age (years)	1.001	[0.988, 1.013]	0.93

CI: confidence interval; SBP_B: mean systolic blood pressure at baseline; Smoking: the proportion of smokers.
‡Significant (P<0.05)

Supplementary Table 7. Moderator estimators for CVD risk in Asia (excluding Indian/South Asian and Iranian populations)

Model	Moderator	e^{β}	95% CI	*P*
Optimal	Sex (1: female; 0: male)	1.528	[1.176, 1.984]	<0.01‡
	Smoking (%)	1.008	[1.003, 1.014]	<0.01‡
Full	Sex (1: female; 0: male)	1.176	[0.528, 2.622]	0.63
	Smoking (%)	1.004	[0.989, 1.018]	0.57
	SBP_B (mmHg)	0.9996	[0.9970, 1.0022]	0.77
	Age (years)	0.994	[0.974, 1.014]	0.47

CI: confidence interval; SBP_B: mean systolic blood pressure at baseline; Smoking: the proportion of smokers.
‡Significant (P<0.05)

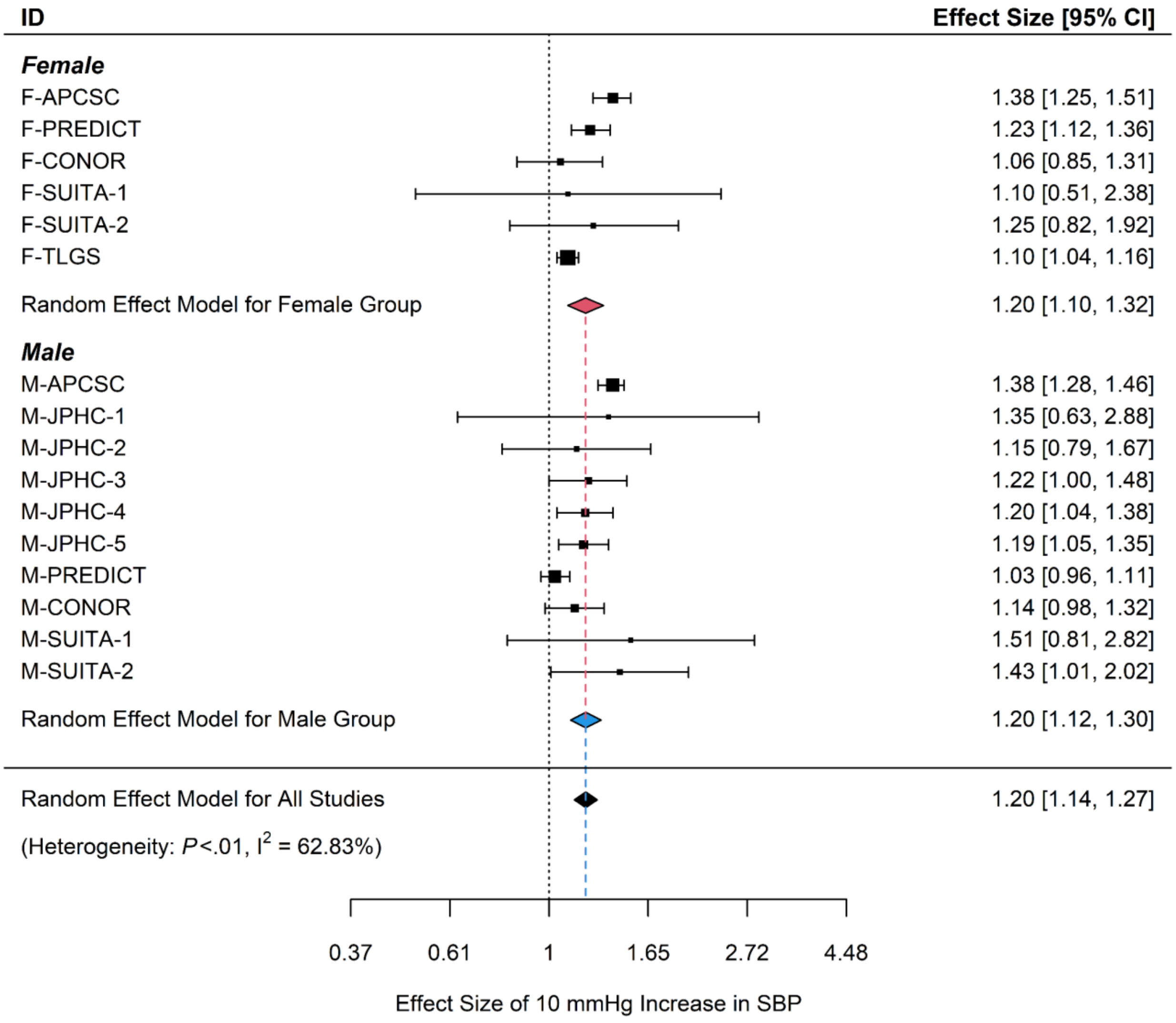

Supplementary Figure 1. Observed and estimated effect sizes for cardiovascular disease risk per each 10-mmHg systolic blood pressure increment in Asia (excluding the SESSA study)

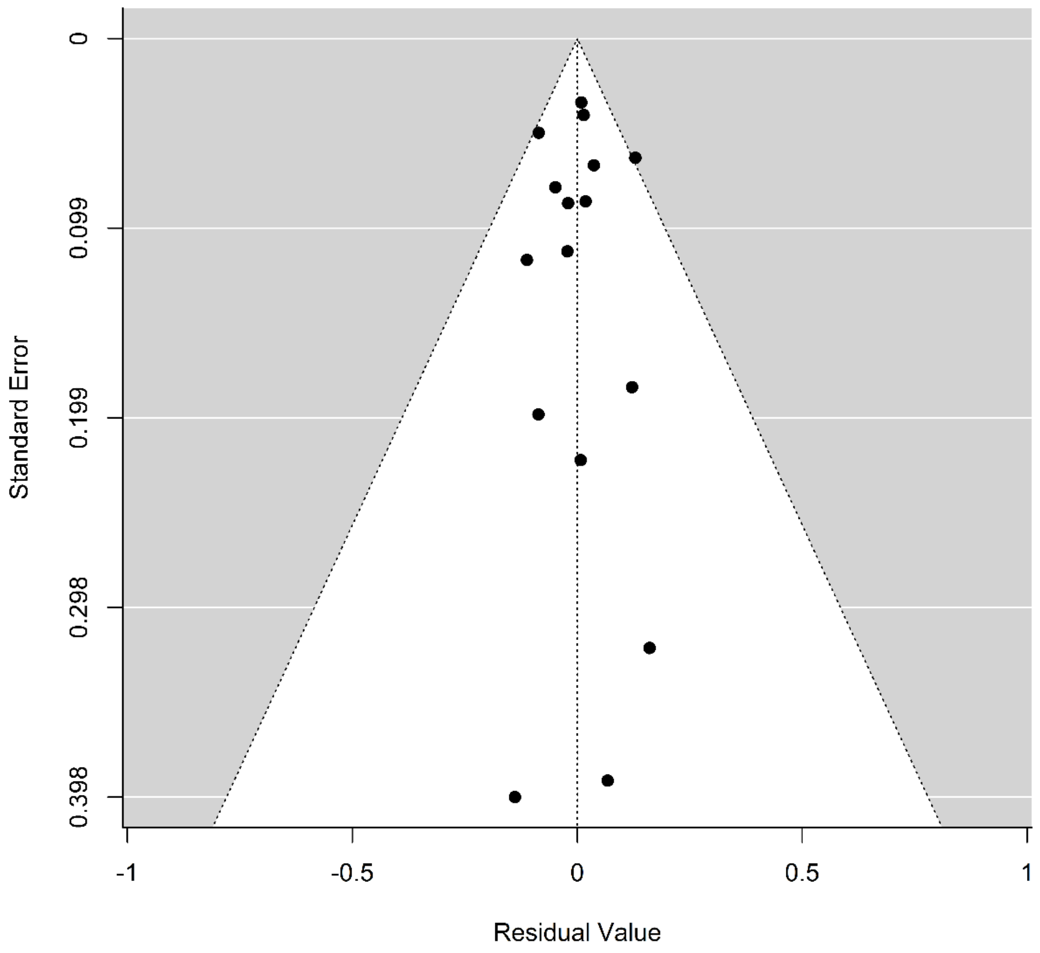

Supplementary Figure 2. Funnel plot of the publication bias for effect sizes in Asia (excluding the SESSA study)

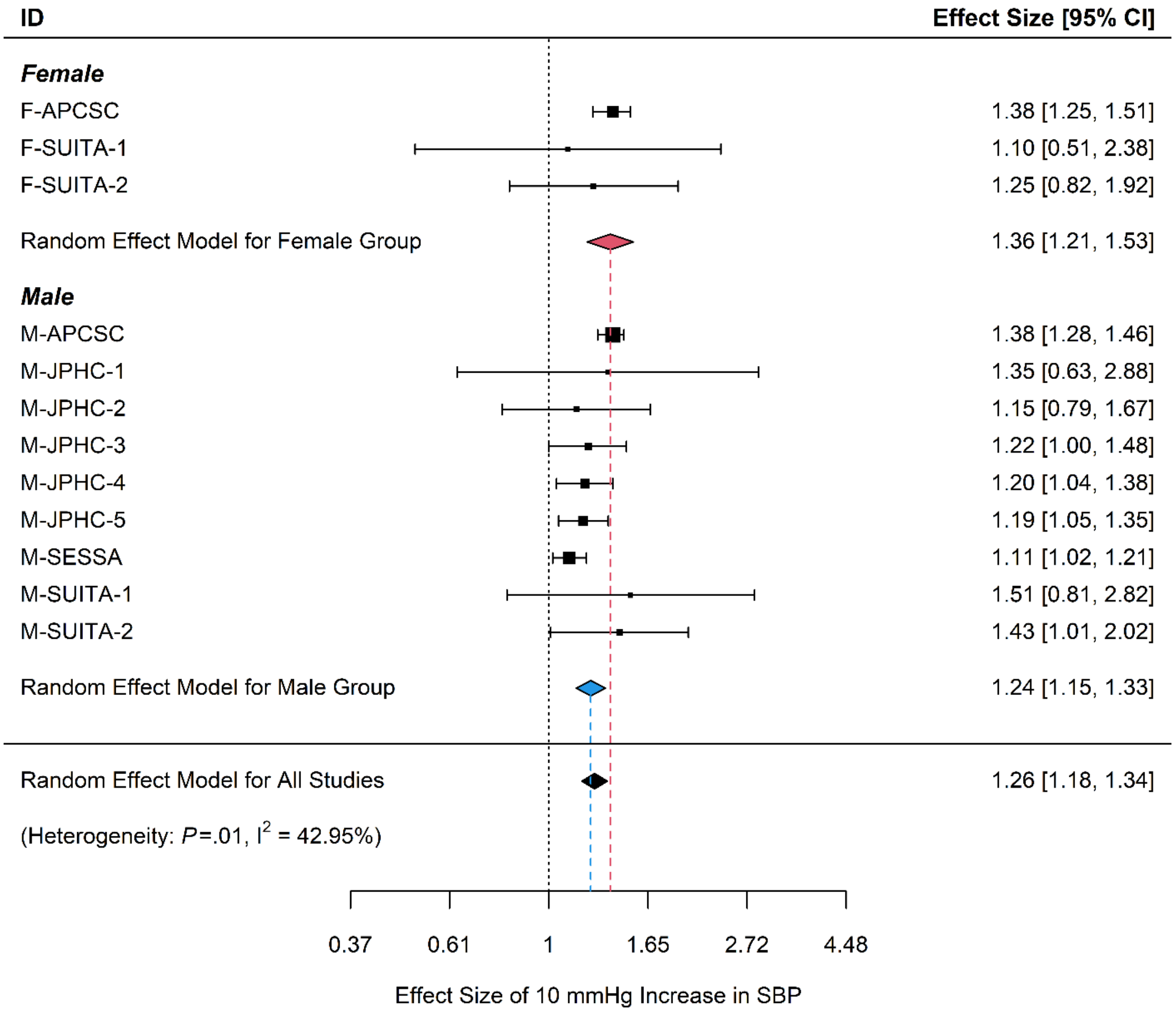

Supplementary Figure 3. Observed and estimated effect sizes for cardiovascular disease risk per each 10-mmHg systolic blood pressure increment in Asia (excluding Indian/South Asian and Iranian populations)

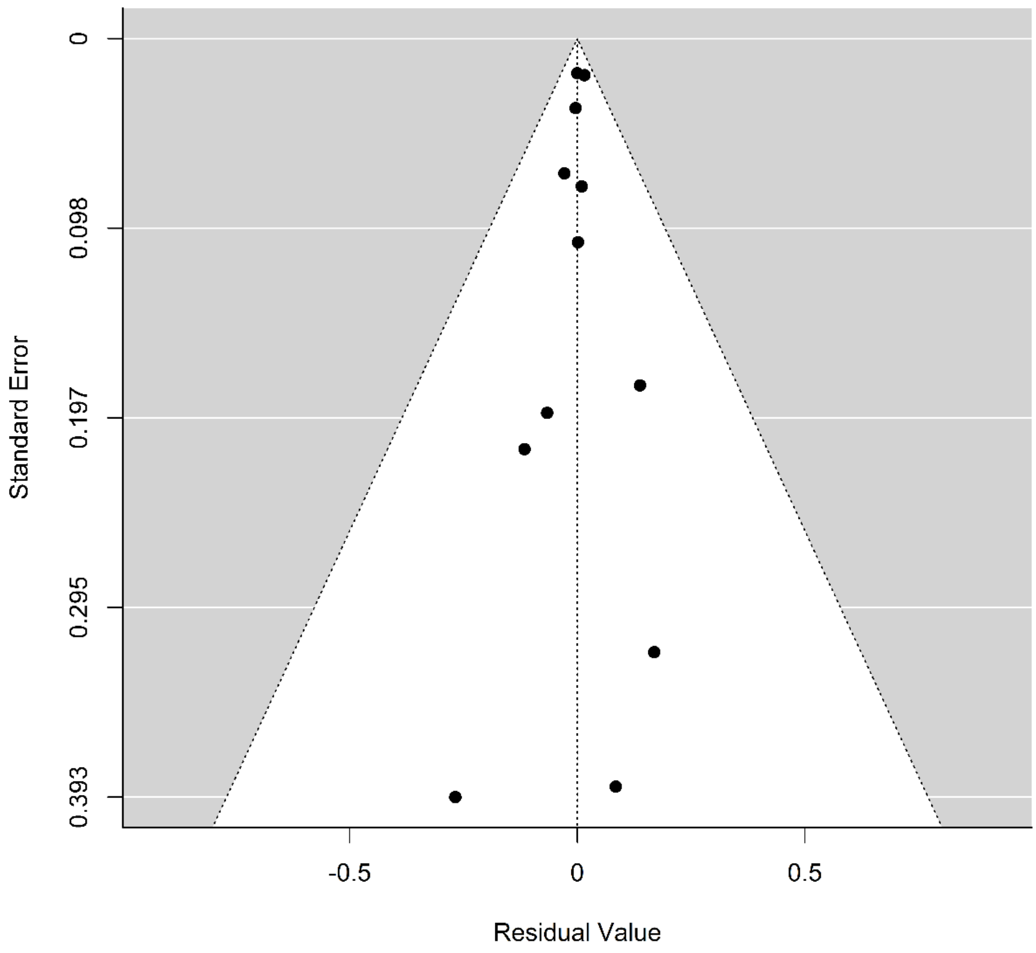

Supplementary Figure 4. Funnel plot of the publication bias for effect sizes in Asia (excluding Indian/South Asian and Iranian populations)

References

1. **Moher D, Liberati A, Tetzlaff J, Altman DG.** Preferred reporting items for systematic reviews and meta-analyses: the PRISMA statement. *International Journal of Surgery*. 2010; 8(5): 336-41. DOI: https://doi.org/10.1016/j.ijsu.2010.02.007
2. **Stroup DF, Berlin JA, Morton SC, Olkin I, Williamson GD, Rennie D,** et al. Meta-analysis of observational studies in epidemiology: a proposal for reporting. *JAMA*. 2000; 283(15): 2008-12. DOI: https://doi.org/10.1001/jama.283.15.2008
3. **Peters SA, Woodward M, Lam TH, Fang X, Suh I, Ueshema H,** et al. Sex disparities in risk and risk factors for ischemic heart disease in the Asia-Pacific region. *European Journal of Preventive Cardiology*. 2014; 21(5): 639-46. DOI: https://doi.org/10.1177/2047487313484689
4. **Rabanal KS, Meyer HE, Tell GS, Igland J, Pylypchuk R, Mehta S,** et al. Can traditional risk factors explain the higher risk of cardiovascular disease in South Asians compared to Europeans in Norway and New Zealand? Two cohort studies. *BMJ Open*. 2017; 7(12): e016819. DOI: https://doi.org/10.1136/bmjopen-2017-016819
5. **Kokubo Y, Kamide K, Okamura T, Watanabe M, Higashiyama A, Kawanishi K,** et al. Impact of high-normal blood pressure on the risk of cardiovascular disease in a Japanese urban cohort: the Suita study. *Hypertension*. 2008; 52(4): 652-9. DOI: https://doi.org/10.1161/HYPERTENSIONAHA.108.118273
6. **Mohebi R, Mohebi A, Sheikholeslami F, Azizi F, Hadaegh F.** Wrist circumference as a novel predictor of hypertension and cardiovascular disease: results of a decade follow up in a West Asian cohort. *Journal of the American Society of Hypertension*. 2014; 8(11): 800-7. DOI: https://doi.org/10.1016/j.jash.2014.08.010
7. **Ikeda A, Iso H, Yamagishi K, Inoue M, Tsugane S.** Blood pressure and the risk of stroke, cardiovascular disease, and all-cause mortality among Japanese: the JPHC Study. *American Journal of Hypertension*. 2009; 22(3): 273-80. DOI: https://doi.org/10.1038/ajh.2008.356
8. **Pham T, Fujiyoshi A, Arima H, Tanaka-Mizuno S, Hisamatsu T, Kadowaki S,** et al. Association of coronary artery calcification with estimated coronary heart disease risk from prediction models in a community-based sample of Japanese men: the Shiga Epidemiological Study of Subclinical Atherosclerosis (SESSA). *Journal of Atherosclerosis and Thrombosis*. 2018; 25(6): 477-89. DOI: https://doi.org/10.5551/jat.42416
